# Investigating the Preservation and Utilization of the Saffron (*Crocus sativus* L.) Sorting By-Product (Tepals)

**DOI:** 10.3390/plants14192949

**Published:** 2025-09-23

**Authors:** Evanthia Dina, Antigoni Cheilari, Dimitra Karamani, Vasilis Mitsopoulos, Ioanna Diamanti, Nikolas Fokialakis, Nektarios Aligiannis

**Affiliations:** 1 Department of Pharmacognosy and Natural Products Chemistry, Faculty of Pharmacy, National and Kapodistrian University of Athens, Panepistimiopolis Zografou, 15771 Athens, Greece; fokialakis@pharm.uoa.gr (N.F.); aligiannis@pharm.uoa.gr (N.A.); 2 Pellas Nature S.A., Seremes, Proastio, 58200 Edessa, Greece; dkaramani@hotmail.com (D.K.); idiama@pellasnature.com (I.D.); 3 Cooperative de Safran, Evripidou 1, 50010 Krokos Kozani, Greece; mitsopoulos@safran.gr

**Keywords:** *Crocus sativus* by-products, tepals, saffron, microwave assisted extraction, adsorption resin, pilot scale

## Abstract

Medicinal and Aromatic Plant (MAP) by-products constitute a vast reservoir of bioactive metabolites with antioxidant and antimicrobial properties, offering potential for the development of high added value natural products. This study focuses on the by-product (tepals) remaining during the process of receiving the stigma of *Crocus sativus* L. Iridaceae (saffron), which is the commercially exploitable part of the flowers. The tepals are the main part of the flowers (>95%) and are still discarded in the fields during the collection of the stigmas in Kozani, Greece. According to numerous findings, the saffron tepals are rich in flavonoids with notable biological properties, and our aim was to investigate an alternative for their management. Within this context, dry and frozen tepals were extracted at a laboratory scale through microwave-assisted extraction (MAE) and ultrasound-assisted extraction (UAE), followed by adsorption resin technology (ART) for the preparation of enriched extracts. Furthermore, their HPTLC profiling, the phenolic (TPC) and flavonoid (TFC) content, and the free-radical-scavenging (DPPH) and anti-tyrosinase activity were evaluated. The frozen tepals were further extracted at a pilot scale using MAE and maceration (Mc) techniques, followed by ART treatment to remove the contained sugars. The enriched extract produced at a pilot scale using MAE and ART sequentially is characterized by a high phenolic (147.2 mg GAE/g dry extract) and flavonoid (114.8 mg QUE/g dry extract) load. These findings demonstrate that saffron tepals, traditionally considered waste, can serve as a valuable raw material for producing extracts rich in phenolic derivatives, particularly flavonoids and anthocyanins.

## 1. Introduction

In recent decades, there has been an ever-increasing consumer preference for products derived from Medicinal and Aromatic Plants (MAPs), which is consistent with their widespread use in the pharmaceutical, cosmetic, and food supplement industry. However, during the processing of MAPs for the development of final products, the unavoidable production of large amounts of by-products and/or wastes has been recorded, which, due to the absence of alternative solutions, are discarded to the environment, causing serious ecological problems [1]. It is therefore critical to make an evaluation of all considered “wastes”, and one of the goals of our research group is to investigate the possibility of their utilization for the development of high added value products [2,3,4]. The Crocus of Kozani, or saffron (*Crocus sativus* L. Iridaceae), is a PDO (Protected Designation of Origin) product of Greece with significant economic value [5]. Cultivations are located in the homonymous region of central Macedonia, and harvesting lasts for a limited period of time (about 1 month). According to the Cooperative of Saffron Producers in Kozani [6], which has the exclusive right to collect, pack, and distribute Kozani saffron, during harvesting, a very large amount of tepals (about 100 tons) are produced. After the sorting of crocus stigmas, the tepals, which cover 95% of the remaining plant parts, are discarded in the fields, and, therefore, the exploitation of this by-product is crucial. *Crocus* species have been extensively studied, and their pharmacological activities have been documented [7,8]. In addition, tepal extracts have demonstrated notable biological activities, such as antihypertensive, antitussive, anti-inflammatory, and antidepressant effects [9]. Moreover, extracts of *C. sativus* L. tepals showed inhibitory effects against alpha-glucosidase and significant antidiabetic properties in an in vitro study [10]. Besides carotenoids, the main components found in crocus extracts from tepals are flavonoids [11,12].

Over the past decade, there has been a systematic effort to replace conventional extraction methods (e.g., infusion, percolation, etc.) with modern, environmentally friendly techniques such as accelerated solvent extraction (ASE), microwave-assisted extraction (MAE), liquid–liquid extraction with centrifugation (ACE), as well as the use of adsorption resin technology (ART) and extraction with aqueous cyclodextrin solutions [13,14,15,16,17,18]. The use of “green extraction” techniques, characterized by lower cost, reduced processing time, and negligible consumption of organic solvents, ensures the recovery of natural products from plant extracts that can be used for the development of innovative food and dietary supplements [19]. One of the objectives of this study was to investigate the possibility of utilizing tepals, which are considered a by-product, for the preparation of extracts with significant antioxidant and potentially anti-aging properties. Various experimental processes were applied on a laboratory and pilot scale to extract flavonoids, based on MAE, UAE, and ART techniques. The study focused on dried and frozen tepals, and the results of the lab-scale extraction processes are presented. The aim was to choose the most suitable method for preparing an extract enriched in phenolic derivatives on a pilot scale, considering the chemical content profiled by HPTLC, the total phenolic (TPC) and flavonoid (TFC) load, as well as the DPPH radical-scavenging capacity and the inhibitory activity against tyrosinase. MAE is an emerging green technique that has been employed in the extraction of natural products [20], and proved to be the method of choice for the pilot-scale extraction of the tepals. Additionally, based on the evaluation of the chemical content of the pilot-scale enriched extract and its ability to scavenge free radicals (DPPH assay), the treatment with ART on a pilot scale was successful. The results revealed that the frozen tepals of *Crocus sativus* L. can be characterized as a valuable raw material from which it is possible to obtain an extract rich in phenolic derivatives, particularly flavonoids and anthocyanins, as shown by LC-MS analysis. To the best of our knowledge, this is the first time that crocus tepals are extracted through MAE at a lab and pilot scale and enriched extracts are developed using the ART technique. In conclusion, this study exposes the urgent need for the utilization of MAP by-products generated in large quantities, since they constitute a vast reservoir of valuable metabolites with significant biological properties, and their exploitation can lead to the development of high added value natural products.

## 2. Results

### 2.1. Lab-Scale Crocus Tepals Extracts

Crocus tepals present issues of stability (sensitivity) at ambient temperature for a period longer than 48 h; hence, after stigma removal, the considered by-product tepals were either immediately frozen or dried in a pilot-scale dryer for better preservation. In order to compare frozen and dried tepals, two extraction methodologies (MAE and UAE) were applied, and the %yield of each extraction was calculated (Table 1).

Based on the data, it is evident that the yields of the extracts produced are similar, whether the MAE or the UAE technique is applied. The only exception is the aqueous extract obtained through UAE (CSUSEdrW, 42.6%), whose yield was clearly higher than the one obtained through MAE (CSMWEdrW, 26.5%). In general, the %yields of the extracts prepared with UAE are slightly higher, but these differences are justified by the variation in extraction time and temperature. Regarding the raw material, the %yields of dried tepals appeared almost 6–7 times higher than the frozen ones because the frozen material carries a large percentage of moisture, and therefore the results are not comparable. It is worth noting that 50 g of frozen tepals after freeze-drying provides 5 g of dry material. Based on the results, and since industrial fridge preservation is more convenient for the producers than drying large amounts of raw material, the frozen material was selected for further pilot-scale investigation.

### 2.2. Processing of Aqueous Crocus Tepal Extracts with ART

All aqueous extracts produced by frozen and dry tepals after MAE and UAE were further processed with adsorption resin technologies (ART) for the development of extracts enriched in flavonoids. Specifically, the methodology of adsorption on long-pore resins was applied to remove sugars and other undesirable water-soluble components to obtain preparations enriched in phenolic derivatives. In Table 2, the codes of the prepared fractions (organic and aqueous), as well as the %yield of the initial charge of the aqueous extracts, are shown.

According to Table 2, the yield of the phenolic fractions of all aqueous extracts (.xadAq) after the treatment with adsorption resins is high (mean value 40.6%) and presents a limited variation (37.3–45.6%). Therefore, the efficiency of the primary aqueous extraction of tepals is the determining factor for the development of an efficient process for obtaining an enriched extract.

### 2.3. Chemical Investigation of Lab-Scale Extracts by HPTLC

**Figure 1:**
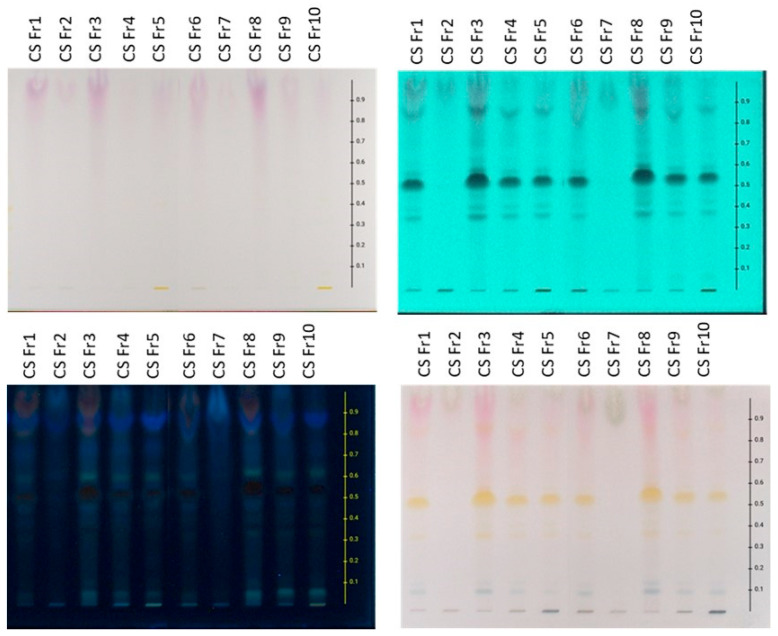
HPTLC chromatograms of frozen tepals extracted with MAE and UAE. Reverse phase HPTLC developed in H_2_O:ACN:A.A/69.31:29.70:0.99. **Upper left**: visible; **upper right**: absorbance at 254 nm; **bottom left**: absorbance at 366 nm; **bottom right**: after spraying with sulfuric vanillin solution and heating.

**Figure 2:**
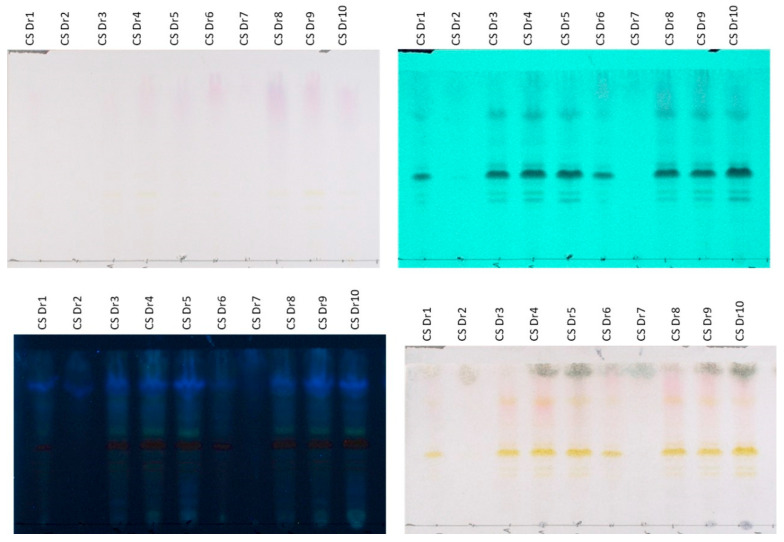
HPTLC chromatograms of dried tepals extracted with MAE and UAE. Reverse phase HPTLC developed in H_2_O:ACN:A.A/69.31:29.70:0.99. **Upper left**: visible; **upper right**: absorbance at 254 nm; **bottom left**: absorbance at 366 nm; **bottom right**: after spraying with sulfuric vanillin solution and heating.

The chemical content of lab-scale extracts, whose coding and description are presented in Table 3, was initially investigated using HPLTC methodology. HPTLC chromatograms obtained from frozen and dried raw materials are presented in Figure 1 and Figure 2, respectively.

Regarding the content of the preparations derived from the frozen plant material, HPTLC chromatograms of extracts prepared using MAE and USE presented similar profiles. In particular, the aqueous (CSFr1, CSFr6), hydroalcoholic (CSFr4, CSFr9), and ethanolic (CSFr5, CSFr10) extracts are characterized by the presence of flavonoids (spots with Rf 0.35–0.65 that show absorbance at 254 and 366 nm and give an orange-yellow color after spraying with sulfuric vanillin), anthocyanins (bands at Rf 0.80–1.00 which are purple when observed at white light, show absorption at 254 and 366 nm, and give a reddish color after spraying with the reagent), sugars (bands at Rf 0.80–1.00 that show no absorption under UV and provide gray-green staining after spraying), and terpenes (bands at Rf 0.00–0.20 with no UV absorbance presenting blue-purple staining after derivatization with sulfuric vanillin reagent). Furthermore, the organic fractions (CSFr3, CSFr8), which were obtained from the aqueous extracts (CSFr1, CSFr6) through ART, showed higher flavonoid and anthocyanin content in comparison with the crude extracts. On the contrary, the zones corresponding to sugars are absent, pointing out the successful application of ART in the enrichment of the extracts. Finally, the aqueous fractions (CSFr2, CSFr7), which were obtained from the same aqueous extracts during the washing step, contain, as expected, mainly sugars.

Likewise, regarding the content of the preparations derived from the dried plant material, extracts prepared with MAE and UAE presented a similar HPTLC chromatographic profile (Figure 2). Specifically, the aqueous (CSDr1, CSDr6), hydroalcoholic (CSDr4, CSDr9), and ethanolic (CSDr5, CSDr10) extracts are characterized by the presence of flavonoids (spots with Rf 0.30–0.65 that absorb at 254 and 366 nm and afford an orange-yellow color after derivatization with sulfuric vanillin), anthocyanins (bands at Rf 0.70–1.00 which are purple when observed at white light, absorb at 254 and 366 nm, and give a reddish color after spraying with the reagent) and sugars (bands at Rf 0.80–1.00 that do not show absorption at 254 and 366 nm and provide a gray-green staining after spraying with sulfuric vanillin). Moreover, the organic fractions (CSDr3, CSDr8), which were obtained from the aqueous extracts (CSDr1, CSDr6) using ART, contain the flavonoids and anthocyanins of the original aqueous extracts, in fact, in a greater concentration. Accordingly, the bands corresponding to sugars are absent. In parallel, the aqueous fractions (CSDr2, CSDr7) obtained from the washing step during the processing with resins contain mainly the sugars of the original aqueous extracts, proving once again the successful application of the adsorbent resin methodology. The results of the HPTLC screening are consistent with the total phenolic load and flavonoid content, which are presented below.

### 2.4. Evaluation of Total Phenolic (TPC) and Flavonoid Content (TFC) in Lab-Scale Extracts

All aqueous, hydroalcoholic, and ethanolic extracts from frozen and dried tepals obtained using MAE and UAE were evaluated for their TPC and TFC content (Table 4). The content of the total phenolic and flavonoid load is expressed as equivalents of GA and QUΕ per gram of dry extract, respectively.

The TPC of extracts obtained with the two extraction techniques (MAE and UAE) was comparable. Particularly, the extracts obtained from frozen and dried tepals showed analogous phenol content. Τhe initial aqueous extracts (CSMWΕfrW, CSMWΕdrW, CSUSEfrW, and CSUSEdrW) presented a similar phenolic load (53.0, 58.0, 61.7, and 61.2 mg GAE/g dry extract, respectively). Likewise, comparable was the phenolic content (67.9, 65.9, 72.3, and 64.8 mg GAE/g dry extract) of the hydroalcoholic extracts (CSMWΕfrW/Et, CSMWΕdrW/Et, CSUSEfrW/Et, and CSUSEdrW/Et, respectively) and the phenolic load (64.4, 60.6, 62.5, and 52.6 mg GAE/g dry extract) of the ethanolic ones (CSMWEfrEt, CSMWEdrEt, CSUSEfrEt, and CSUSEdrEt, respectively). Regarding the organic fractions of MAE aqueous extracts processed with ART (CSMWΕfrWxadOrg and CSMWΕdrWxadOrg), they showed a higher phenolic load (119.9 and 120.3 mg GAE/g dry extract, respectively) compared to the TPC (53.0 and 58.0 mg GAE/g dry extract) of the corresponding initial aqueous extracts (CSMWΕfrW and CSMWΕdrW). On the other hand, the organic fractions of UAE aqueous extracts processed with ART (CSUSEfrWxadOrg and CSUSEdrWxadOrg) exhibited a lower phenolic load (83.0 and 75.1 mg GAE/g extract, respectively) in comparison to MAE ones. Lastly, all aqueous fractions (CSMWΕfrWxadAq, CSMWΕdrWxadAq, CSUSEfrWxadAq, and CSUSEdrWxadAq), which resulted from the treatment of the initial aqueous extracts (CSMWΕfrW, CSMWΕdrW, CSUSEfrW, and CSUSEdrW, respectively) with adsorption resins, showed a very low phenolic content (14.0, 12.2, 18.6, and 6.9 mg GAE/g extract, respectively).

Likewise, similar conclusions were drawn from the study of the TFC content. More specifically, extracts obtained using MAE and UAE, both frozen and dried, exhibited a similar content in flavonoids. The initial aqueous extracts (CSMWΕfrW, CSMWΕdrW, CSUSEfrW, and CSUSEdrW) showed comparable TFC (27.7, 36.9, 22.7, and 25.4 mg QUE/g dry extract, respectively) values. Also, the flavonoid content (30.4, 36.8, 36.2, and 38.0 mg QUE/g dry extract) of the hydroalcoholic extracts (CSMWEfrW/Et, CSMWEdrW/Et, CSUSEfrW/Et, and CSUSEdrW/Et, respectively) and the flavonoid load (42.4, 36.6, 42.8, and 32.6 mg QUE/g dry extract) of the ethanolic extracts (CSMWEfrEt, CSMWEdrEt, CSUSEfrEt, and CSUSEdrEt, respectively) were equivalent between the two extraction methods (MAE/UAE) and the type of the raw plant material (frozen/dry). Additionally, the organic fractions CSMWΕfrWxadOrg and CSMWΕdrWxadOrg obtained using ART from the MAE extracts showed a higher flavonoid load (100.9 and 112.3 mg GAE/g extract, respectively) compared to the load (27.7 and 36.9 mg GAE/g extract) of the corresponding initial aqueous extracts (CSMWΕfrW and CSMWΕdrW). On the contrary, the organic fractions CSUSEfrWxadOrg and CSUSEdrWxadOrg obtained using ART from the UAE extracts presented limited flavonoid load (69.0 and 49.8 mg GAE/g extract, respectively). Finally, as expected, all aqueous fractions (CSMWΕfrWxadAq, CSMWΕdrWxadAq, CSUSEfrWxadAq, and CSUSEdrWxadAq), which resulted from the treatment of the initial aqueous extracts (CSMWΕfrW, CSMWΕdrW, CSUSEfrW, and CSUSEdrW, respectively) with adsorption resins, showed no presence of flavonoids. Regarding the alcoholic extracts, data are comparable to the literature, with values of TPC and TFC ranging from 54.59 to 65.34 and 34.23 to 60.64, respectively [21].

### 2.5. Evaluation of the DPPH Scavenging Capacity in Lab Scale

The antioxidant activity against the DPPH free radicals was evaluated at two concentrations (200 and 100 µg/mL), and according to the results (Table 5) all extract preparations showed moderate to weak activity at 200 μg/mL, scavenging the DPPH free radicals in a percentage of less than 40%. Nevertheless, the highest antioxidant activity was demonstrated by the phenol-enriched extracts obtained using ART (CSMWΕfrWxadOrg, CSMWΕdrWxadOrg, CSUSEfrWxadOrg, and CSUSEdrWxadOrg), as the DPPH free radical inhibition was 36.7, 35.8, 37.0, and 31.6%, respectively.

### 2.6. Evaluation of the Tyrosinase Inhibitory Activity in Lab Scale

The evaluation of the inhibitory effect against the tyrosinase enzyme was carried out, and it was found that all the preparations were inactive, as at a concentration of 300 µg/mL they showed a very low inhibitory effect.

### 2.7. Pilot-Scale Crocus Tepal Preparations Obtained Through MAE, Mc, and ART

Based on the HPTLC chemical profile and the % yields of the lab-scale extracts, the aqueous preparations seemed similar to the hydroalcoholic and ethanolic ones. Therefore, it was decided to use water as the solvent for the preparation of the initial extracts at the pilot scale using MAE and maceration (Mc). In the case of MAE, based on the observations from the preliminary experiments at the lab scale, the solvent/plant material ratio, the temperature, and the extraction duration played a significant role, and, hence, different combinations of these parameters were tested prior to the extraction. According to the protocol of MAE at the pilot scale, 10 kg of frozen tepals were extracted with 30 L of H_2_O for 45 min until the temperature reached 50 °C, while a resting step (10 min) followed. Then, 100 mL of the extract was evaporated to dryness, and the extraction yield was calculated to be 4.1%. In parallel, in the case of Mc, the tepals were extracted with water (10 kg of frozen tepals were mixed with 100 L of H_2_O) in a stainless-steel extractor, and the extraction yield was calculated to be 4.6%. The aqueous extracts obtained using MAE (CSMWEfrWPL) and Mc (CSMcfrWPL) were treated with adsorption resins for the development of extracts enriched in flavonoids, and the % yield of the phenolic fraction was calculated at 37.3 and 44.4%, respectively.

### 2.8. Chemical Investigation of Crocus Tepals Extracts at Pilot Scale by HPTLC

The chemical fingerprint of pilot-scale extracts was investigated and compared with the corresponding lab-scale preparations using HPTLC analysis. Coding of the samples is provided in Table 6.

**Figure 3:**
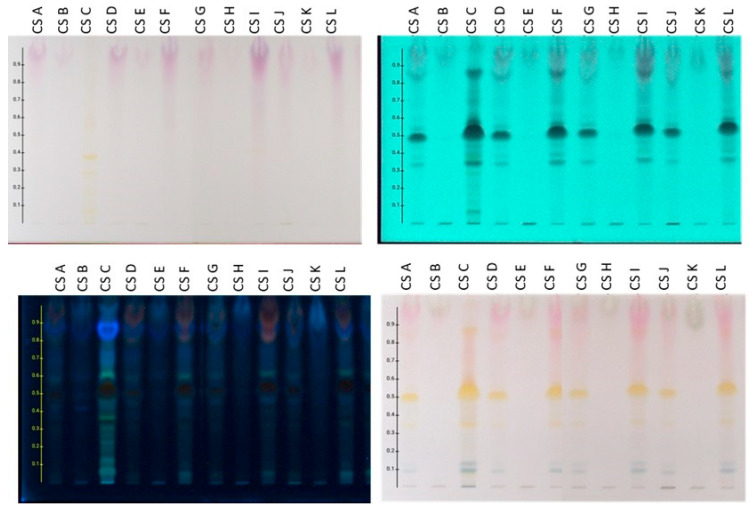
HPTLC chromatograms of frozen tepals extracted using MAE and MC at pilot and lab scale. Reverse phase HPTLC developed in H_2_O:ACN:A.A/69.31:29.70:0.99. **Upper left**: visible; **upper right**: absorbance at 254 nm; **bottom left**: absorbance at 366 nm; **bottom right**: after spraying with sulfuric vanillin solution and heating.

Profiling of all extracts was performed using RP HPTLC methodology, and the resulting chromatograms are shown in Figure 3.

Based on the comparison results of HPTLC profiles for the preparations derived from the frozen plant material prepared on a laboratory and pilot scale, the following conclusions were reached: (a) The extracts obtained using MAE (lab and pilot scale), UAE (lab scale), and Mc (pilot scale) showed a similar chromatographic profile. (b) The initial aqueous extracts (CSA, CSD, CSG, CSJ) are characterized by the presence of flavonoids (mainly spots with Rf 0.30–0.60 absorbing at 254 and 366 nm and showing an orange-yellow color after spraying with sulfuric vanillin reagent), anthocyanins (bands at Rf 0.80–1.00 which are purple when observed under white light, absorb at 254 and 366 nm, and give a reddish stain after spraying with the reagent), sugars (bands at Rf 0.80–1.00 without absorption under UV and exhibiting gray-green color after derivatization with sulfuric vanillin), and terpenes (bands at Rf 0.00–0.20 which are colored blue-purple after spraying with the reagent). (c) The organic fractions (CSC, CSF, CSI, CSL), which were obtained from the aqueous extracts (CSA, CSD, CSG, CSJ) after treatment with ART, contain the flavonoids and anthocyanins of the crude aqueous extracts in higher concentrations. Also, the zones corresponding to sugars are absent. (d) Accordingly, the aqueous fractions (CSB, CSE, CSF, CSK), which resulted from the washing step of the resin treatment, contain mainly the sugars of the original aqueous extracts, verifying the successful application of ART.

### 2.9. Evaluation of Total Phenolic (TPC) and Flavonoid Content (TFC) in Pilot-Scale Extracts

The aqueous extracts prepared on a pilot scale, as well as the organic and aqueous fractions obtained after the treatment with adsorption resins, were evaluated for their TPC and TFC content, and results are shown in Table 7.

Based on the results, it is evident that the aqueous extracts prepared on a pilot scale from frozen tepals using MAE (CSMWEfrWPL) and Mc (CSMcfrWPL) demonstrate a rich phenolic load (65.2 and 59.9 mg GAE/g dry extract) and noticeable flavonoid content (32.6 and 21.4 mg QUE/g dry extract). The organic fractions (CSMWΕfrWPLOrg, CSMcfrWPLOrg) obtained from the aforementioned aqueous extracts have 2–2.5 times higher phenolic load (147.2 and 152.8 mg GAE/g dry extract) and about 4 times higher flavonoid content (114.8 and 85.9 mg QUE/g dry extract). As expected, the corresponding aqueous fractions (CSMWEfrWPLAq, CSMcfrWPLAq) present a very poor content of phenols (16.5 and 12.4 mg GAE/g dry extract) and no flavonoid content. Based on these results, we present herein that the tepals of the *Crocus sativus* L. species are a valuable raw material, from which it is possible to obtain an extract rich in phenolic derivatives and especially in flavonoids and anthocyanins.

### 2.10. Evaluation of the DPPH Scavenging Capacity at Pilot Scale

The aqueous extracts prepared on a pilot scale using MAE and Mc, as well as the organic and aqueous fractions obtained after the treatment with resins, were evaluated for their ability to scavenge DPPH free radicals. The antioxidant activity was evaluated at concentrations of 200 and 100 µg/mL, and the results were expressed as % inhibition of DPPH free radicals (Table 8).

According to the results, all preparations showed moderate to low activity at a concentration of 200 µg/mL, as they inhibited the DPPH free radicals at a percentage of less than 50%. Nonetheless, the highest antioxidant activity was demonstrated by the phenol-enriched preparations CSMWΕfrWPLOrg and CSMcfrWPLOrg, as they inhibited the DPPH free radicals by 47.5 and 37.3%, respectively, at a concentration of 200 μg/mL.

### 2.11. Evaluation of the Tyrosinase Inhibitory Activity at Pilot Scale

From the evaluation of the inhibitory effect against the enzyme tyrosinase of pilot-scale preparations, it was concluded that all extracts were inactive, as at a concentration of 300 µg/mL they showed no inhibition.

### 2.12. UHPLC-HRMS Analysis

The chemical profiling of the enriched extracts obtained using MAE at a lab and pilot scale was also performed by LC-MS. The annotation of the compounds is presented in Table 9, and was achieved by comparing the experimental mass measurements and the corresponding theoretical ones (mass difference expressed in ppm). Moreover, the proposed molecular formulas, together with the Ring–Double bond equivalent combined with data from the literature [11,22] and mass databases such as FOODB [23] and Dictionary of Natural Products [24], allowed the annotation of 40 compounds.

The data in Table 9 agree with the results of the preliminary control of the content by HPTLC. More specifically, the extracts of the by-product (tepals) of the saffron sorting process mainly contain (a) polyphenols (tetrahydroxyflavone diacetyl-coumaroyl hexoside, biflavanol hexoside, tetrahydroxy-flavone diglucoside, lignan glycoside, methyl-anthocyanidin coumaroyl glucoside, flavonol trihexoside, tetrahydroxy flavone trihexoside, trihydroxy flavone gallate, tetrahydroxy flavone acetyl dihexoside, hexahydroxyflavone dihexoside, tetrahydroxy flavone malonyl dihexoside, tetrahydroxyflavone glucoside, tetrahydroxyflavone diglucoside, tetrahyhydroxy-dimethoxy flavone diglucoside, trihydroxyflavonol dihexoside, tetrahydroxyflavone dihexoside, trixydroxy-methoxy flavone dihexoside, galloylglucosyl acetophenone, (epi)catechin glucuronide, tetrahydroxy flavonol malonylglucoside, tetrahydroxy flavonol acetylglucoside, and tetrahydroxyflavanone), (b) phenolic compounds (cinnamoyl hexoside, methyl-galloyl glucoside, phenolic dihexoside, coumaroyl glucosides, hydroxyphenyl hexosides, and acetophenone galloylglucosides), (c) carotenoids (crocin 2 and crocin 3), and (d) sugars (hexoses, disaccharides, trisaccharides, bis-pentoses, and bis-hexoses).

### 2.13. Statistical Analysis

**Figure 4:**
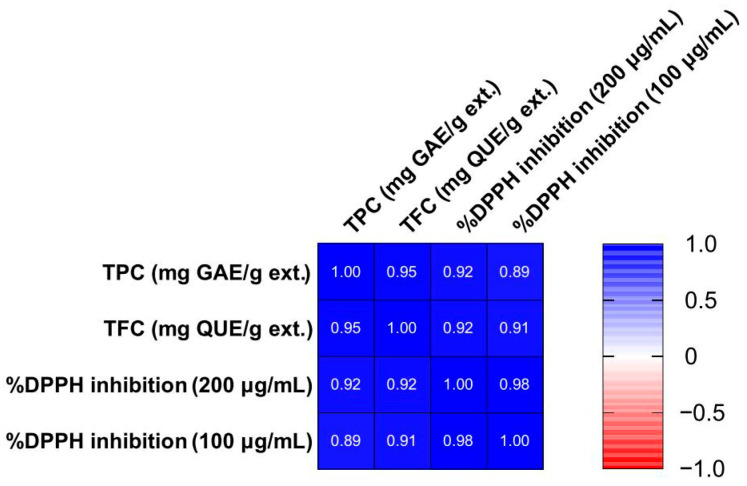
Tepal extract TPC and TFC content correlate with DPPH inhibition activity. Blue color represents positive Pearson’s correlation coefficients (*p* < 0.0001 for all correlations).

**Figure 5:**
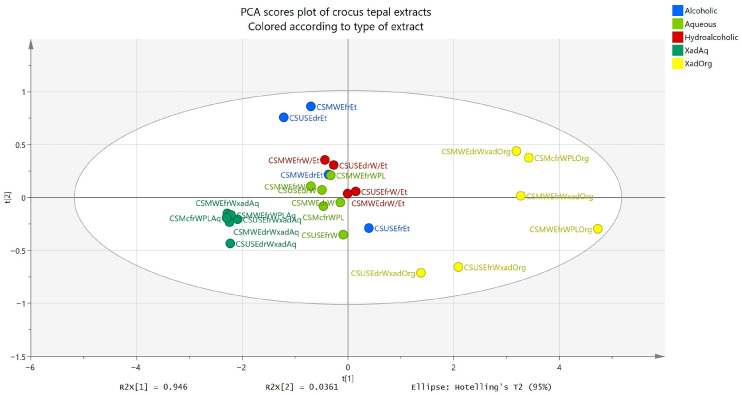
PCA score scatter plot of tepal extracts originating from MAE (lab and pilot scale), UAE (lab scale), and Mc (pilot scale) extraction procedures, and after treatment with ART. Observations are colored according to the type of extract.

To better visualize the results and examine how the three tests (TPC, TFC, DPPH at 200 μg/mL and 100 μg/mL) are related, a correlation matrix was constructed (Figure 4) using Pearson correlation. All correlation coefficients (r) were higher than 0.89, with *p* < 0.0001. As expected, extracts richer in phenols and flavonoids exhibited stronger DPPH inhibition. Complementarily, a PCA model was constructed using TPC, TFC, and DPPH values as variables. The PCA included two principal components (PCs) which together accounted for 98.2% of the explained variance (R^2^X(cum) = 0.982, Q^2^(cum) = 0.927). As shown in Figure 5, the phenol-enriched extracts obtained after ART treatment clustered closely together, regardless of whether they originated from MAE (lab and pilot scale), UAE (lab scale), or Mc (pilot scale) aqueous extracts. Accordingly, the aqueous, the hydroalcoholic, and the ethanol extracts formed distinct groups, while the fractions from the washing step after ART were more widely dispersed. The organic and aqueous fractions derived from ART treatment showed the greatest separation along PC1 as expected.

## 3. Discussion

In conclusion, we could assert that the development of high added value natural products through the exploitation of *Crocus sativus* L. tepals, which are considered as a by-product of the saffron sorting process, is feasible. In particular, the production on an industrial scale for extraction using the MAE technique, followed by treatment with adsorption resins, is promising and can afford a preparation rich in flavonoids and other phenolic compounds. To the best of our knowledge, this is the first systematic comparison study of crocus tepal extracts at lab and pilot scales using chromatomeric (TPC, TFC) and chromatographic (HPTLC, LC-MS) techniques, as well as the evaluation of the %DPPH inhibition. Specifically, this study exhibits that the large quantities of the by-product that are produced during the processing of saffron raw material constitute a huge reservoir of valuable metabolites with important biological properties (e.g., antioxidants). Hence, it is imperative to be exploited for the development of final products of high added value and to be utilized in the sectors of phytotherapeutics, herbal medicines, cosmeceuticals, nutraceuticals, and food supplements, rather than being treated as waste and discarded in the environment. In general, the activities of the agri-food sector produce large quantities of liquid and solid waste (industrial by-products), polluting the soil due to high organic matter content. We propose herein the treatment of tepal extracts with ART in order to recover the organic load to obtain enriched extracts in phenolic compounds. This proposal aims to encourage saffron producers to implement a zero-waste strategy in the case of this agro-industrial waste (tepals) and develop bioactive natural products that can be used as active ingredients in food, beverages, and food supplements. However, the consolidation of this process needs further consideration in terms of qualitative and quantitative composition of both the raw material and the extract every year. Thus, for saffron producers, the assessment of a techno-economic analysis investigating the utilization of tepal-enriched extract based on its production cost and commercial value is crucial, since it is obvious that this non-tradeable plant material is a valuable source of bioactive compounds.

## 4. Materials and Methods

### 4.1. Chemicals, Standards, and Materials

Ethanol (EtOH) for extractions, acetonitrile (ACN), acetic acid (AA), sulfuric acid (H_2_SO_4_), and vanillin for HPTLC analysis, ethanol 96% (EtOH) for bioassays, and potassium hydroxide (KOH) were purchased from Merck (Merck, Darmstadt, Germany). Distilled water was produced from LaboStar Pro TWF UV ultra-pure water system (Evoqua Water Technologies, Barsbuettel, Germany). For free radical scavenging and total phenolic content assays, Folin–Ciocalteu solution, dimethylsulfoxide (DMSO), sodium carbonate (Na_2_CO_3_), aluminum chloride (AlCl_3_), sodium acetate (CH_3_COONa), gallic acid (GA), quercetin (QUΕ), and 2,2-diphenyl-1-picrylhydrazyl (DPPH) were purchased from Sigma-Aldrich (Sigma-Aldrich, Steinheim, Germany).

### 4.2. Plant Material and Sampling

Crocus tepals were collected in collaboration with the farmers of the Kozani Saffron Producers Cooperative in Greece, where the plant is cultivated. During the harvest season (October–November), crocus tepals were collected after sorting and receiving the stigmas. After stigma removal, the considered by-product tepals were either immediately frozen or dried. In particular, the tepals were dried in a pilot-scale dryer for better preservation, and, in parallel, 100 kg were kept immediately after sorting the crocus stigmas in an industrial deep freezer (−80 °C) until the experiments were carried out for the pilot production of the enriched extracts. The samples of the two different elaboration processes (frozen and dried) were sent to the Laboratory of Pharmacognosy and Natural Products Chemistry for the lab-scale study. Samples were transferred in a special dry ice package for better preservation and stored. Voucher specimens are kept in the Lab of Pharmacognosy and Natural Products Chemistry (EDCSt001d, EDCSt001f for dried and frozen material, respectively).

### 4.3. Preparation of Extracts from Crocus Tepals (Frozen, Dried)

#### 4.3.1. Lab-Scale Microwave-Assisted Extraction (MAE)

A Milestone NEOS-GR microwave unit (Milestone Srl, Sorisole, Italy) was used for the extraction of raw material. All experiments were performed with a method including a 10 min step-rising time at 70 °C and a 20 min holding time at the same temperature. The extraction was performed using microwave irradiation at a power of 900 W. Three different solvents were selected for the extraction of the raw plant material (frozen and dried). Particularly, 100 g of frozen and 50 g of dried tepals (the volume of dry tepals was almost twice the volume of the frozen ones) were extracted separately with water (1 L H_2_O), hydroalcoholic mixture (1 L H_2_O/EtOH 50/50), and ethanol (1 L EtOH). After the completion of the extraction, the mixtures were left to cool down, filtered, and dried by the solvents’ removal under reduced pressure using a rotary evaporator (Büchi Labor-technik AG, Flawil, Switzerland). The six extracts were stored at 4 °C until analysis. In the case of aqueous extracts, a part was kept at −10 °C for further processing with adsorption resins.

#### 4.3.2. Pilot-Scale MAE

The pilot production experiments took place at the facilities of the company “Pellas Nature”, which is based in Edessa, Greece, and is active in the production of flavored olive oils with the application of MAE. The extractions were carried out in a pilot microwave extraction device, the Milestone NEOS-GR microwave unit (Milestone Srl, Sorisole, Italy), carrying a 50 L extraction chamber. The frozen plant material was transferred from the storage area to the company (85 Km) on the same day that the extractions took place. A total of 10 kg of crocus tepals was extracted with 30 L H_2_O for 55 min (45 min step-rising time at 50 °C and 10 min holding time). The extraction was performed using microwave irradiation at a power of 6000 W, while the pressure during the extraction was 330 mbar. After the chamber’s cooling down, the extraction basket was removed, and the aqueous extract was received after filtration. Part of the aqueous extract was dried for %yield calculation, and the remaining was stored at −10 °C for further processing (adsorption resins).

#### 4.3.3. Lab-Scale Ultrasound-Assisted Extraction (UAE)

In the general context of the evaluation of the chemical load of the raw materials (frozen and dried tepals), aqueous, hydroalcoholic, and ethanolic extracts were produced using the Elma S 100 H ultrasound-assisted extraction (UAE) apparatus (Elmasonic, Singen, Germany). For each plant material, three different extractions were performed. Specifically, 20 g of frozen and 10 g of dried tepals were extracted with 200 mL of each solvent medium (H_2_O, H_2_O/EtOH 50/50, EtOH) for a total of 30 min (three extraction cycles) at 40 °C. The six extracts were filtered, dried, and stored at 4 °C until analysis. Again, in the case of aqueous extracts, a part was kept at −10 °C for further processing with adsorption resins.

#### 4.3.4. Pilot-Scale Extraction Through Maceration

In order to compare the pilot-scale MAE with a conventional extraction process, the frozen tepals were extracted through maceration (Mc) in a stainless-steel extractor in the area of Kozani. More specifically, 10 kg of frozen tepals was mixed with 100 L of H_2_O and left while stirring (using a fan) at regular intervals (10 min of stirring every 4 h) for 48 h. The extract was filtered and stored at −10 °C for further processing, while an aliquot was dried for %yield calculation.

### 4.4. Development of Flavonoid-Enriched Extracts Using Adsorption Resin Technology (ART)

All aqueous extracts (lab and pilot scale) were processed with XAD-4 type macroporous polystyrene Amberlite FPX66 (Rhom and Haas, Philadelphia, PA, USA) resin, with surface area 750 m^2^/g, porosity ≥ 0.50 m/mL, particle size 0.3–1.2 mm, and pore envelope 55–80 A°. According to the protocol applied, 200 mL of each aqueous extract was treated with 50 mL of resin (ratio 4:1) using the bath technique. More specifically, the aqueous solution and activated resin were placed in a conical flask and left under magnetic stirring at room temperature for 2 h (adsorption stage). After the completion of the adsorption stage, the resin was filtered and washed with 200 mL of H_2_O to remove the unadsorbed substances. Then, the phenolic derivatives were released from the resin with 100 mL of EtOH. The extracts enriched in flavonoids were obtained after EtOH removal in a rotary evaporator. Samples were stored at 4 °C until analysis.

### 4.5. High-Performance Thin-Layer Chromatography (HPTLC) Profiling

The chemical profile of the obtained extracts was determined using a High-Performance Thin-Layer Chromatography (HPTLC) apparatus purchased from CAMAG (Muttenz, Switzerland). Samples were applied on reversed phase (RP) plates precoated with silica gel 60 RP-18 F_254S_ (Merck, Darmstadt, Germany) using an automated sample applicator ATS4, and the chromatograms were developed in an ADC2 automated development chamber with the appropriate mobile phase. The plates were documented under UV 254 and 366 nm, and after spraying with sulphuric vanillin and heating using the TLC Visualizer 2. The system was operating under the VisionCats 2.2 software. For the HPTLC fingerprinting, 100 μg of each sample was loaded on the RP plates, and solvent mixtures of H_2_O:ACN:A.A (69.31:29.70:0.99) were used as a mobile phase.

### 4.6. Total Phenolic Content (TPC) Determination

The phenolic content of the extracts was determined by the Folin–Ciocalteu colorimetric method [25]. Folin–Ciocalteu solution was prepared with 10% dilution in distilled water, and the alkaline environment was achieved with the addition of 7.5% sodium carbonate in distilled water. Extracts were prepared using DMSO as a solvent in stock concentrations, and dilutions were made if necessary. In 96-well plates, 25 µL of extract in DMSO, 125 µL Folin–Ciocalteu solution, and 100 µL Na_2_CO_3_ solution were mixed. The plates were incubated for 30 min at ambient temperature in the dark. Absorbance was measured at 765 nm using a microplate reader (Infinite M200 PRO, Tecan, Männedorf, Switzerland). The TPC of the extracts was determined by a standard curve of absorbance values derived from standard concentration solutions of GA (1.25, 2.5, 5, 10, 20, 30, 40, 50, and 100 µg/mL final concentrations). Extracts were initially tested at a concentration of 100 µg/mL (final concentration in the well), and those that exhibited absorbance value outside of the linear part of the curve were measured again in a higher or lower concentration. TPC was expressed as mg of GA equivalents per g of dried extract (mg GAE/gr dry weight), and each sample was tested in triplicate.

### 4.7. Total Flavonoid Content (TFC) Determination

The total flavonoid content (TFC) of all extract samples was estimated using the aluminum chloride method. AlCl_3_ solution was prepared with 1.8% *w*/*v* dilution in distilled water, and the alkaline environment was achieved with the addition of sodium acetate 1 M in distilled water. Extracts were prepared using DMSO in stock solution, and dilutions were made if necessary. In 96-well plates, 50 μL of extract in DMSO, 20 μL aluminum trichloride hydrate solution, 160 μL ethanol absolute, and 20 μL sodium acetate were mixed. The reaction mixture was incubated for 40 min in ambient temperature in the dark, and absorbance was measured at 415 nm in a microplate reader. TFC was estimated from a standard curve of absorbance values derived from standard concentration solutions of QUΕ. Seven serial dilutions (12.5, 25, 50, 100, 200, 400, and 800 μg/mL final concentrations) were prepared from a stock solution of 10 mg/mL in DMSO. Extracts were initially tested at the concentration of 800 μg/mL (final concentration in the well), and those that exhibited absorbance values outside of the linear part of the curve were measured again in lower concentrations. TFC in tested samples was determined based on a linear regression equation of standard curve and expressed in QUΕ equivalents (mg QUE/g).

### 4.8. DPPH Scavenging Activity Assay

Evaluation of the antioxidant activity of the produced hydro-alcoholic and aqueous extracts was performed using the free radical 2,2-diphenyl-1-picrylhydrazyl (DPPH) assay as described previously [26]. Extracts were prepared using DMSO as a solvent in an initial concentration of 4 mg/mL (stock solution), and dilutions were made, if necessary, to reach the tested concentrations. A total of 10 μL of extract in DMSO and 190 μL of DPPH solution (12.4 mg/100 mL in ethanol) were mixed in a 96-well plate and then subsequently incubated at room temperature for 30 min in darkness. Finally, the absorbance was measured at 517 nm in a microplate reader. All evaluations were performed in triplicates. GA was used as positive control, and the % inhibition of the DPPH radical was estimated by the following equation: [(A − B) − (C − D)]/(A − B) × 100, where A: control (without sample), B: blank (without sample, without DPPH), C: sample, D: blank sample (without DPPH).

### 4.9. Tyrosinase Inhibitory Activity

The ability of the extracts to prevent the oxidation of L-DOPA to dopaquinone and subsequently to dopachrome by mushroom tyrosinase was evaluated at a concentration of 300 μg/mL. In 96-well plates were placed 40 µL of sample, 80 µL buffer solution (pH = 6.7 ± 0.02), and 40 µL tyrosinase (92 u/mL) dissolved in buffer. Tyrosinase from mushroom lyophilized powder, ≥1.000 unit/mg solid T3824 (25KU), was purchased from Sigma-Aldrich (Sigma-Aldrich, Steinheim, Germany). For each sample, blanks were used which did not contain tyrosinase, and a control sample was created (120 µL of buffer solution and 40 µL of tyrosinase). Kojic acid (IC50 = 2 μg/mL) and the methanolic extract of licorice root (IC50 = 5 μg/mL) were used as standard inhibitors. All sample measurements were performed in triplicates after incubation for 5 min at 25 °C, and the absorbance was measured at 475 nm using a microplate reader. Tyrosinase inhibition was calculated by the following formula: {[(A − B) − (C − D)]/(AB)} × 100. A: control (without sample), B: blank (without sample and tyrosinase), C: sample, D: blank of the sample (without tyrosinase).

### 4.10. Ultra-High-Performance Liquid Chromatography—High-Resolution Mass Spectrometry (UHPLC-HRMS) Analysis

The ultra-high-performance liquid chromatography was performed employing a Accela UHPLC system (Thermo Scientific, Darmstadt, Germany) equipped with a binary pump, an autosampler, an online vacuum degasser, and a temperature-controlled column compartment. LC-MS grade methanol (MeOH) and formic acid (FA) were purchased from Fisher Scientific (Fisher Optima, Loughborough, UK). An Ascentis C18 150 × 2.1 mm, 3 μm reversed phased column (Supelco Analytical, Bellefonte, PA, USA) was used for the analysis. The high-resolution mass spectrometry was performed on a LTQ Orbitrap Discovery XL mass spectrometer (Thermo Scientific, Germany). Samples were injected at concentration of 100 ppm diluted in MeOH:H_2_O 50:50. The mobile phase consisted of solvents A: aqueous 0.1% (*v*/*v*) formic acid and B: acetonitrile. Different gradient elutions were performed for positive and negative ion mode detection, and after optimization of the chromatography the gradient applied was T = 0–3 min, 5% B; T = 3–24 min, 5% B. The flow rate was 0.4 mL/min and the injection volume was 5 μL, while the column temperature was kept at 40 °C. The ionization was performed at HESI, with both positive and negative modes. The conditions for the HRMS were set as follows: capillary temperature, 300 °C; spray voltage, 3.3 kV; S-lense Rf level, −100 V; capillary voltage, −35 V; sheath gas flow, 40 arb. units; aux gas flow, 10 arb. units; aux. gas heater temperature, 50 °C. Analysis was performed using the Fourier transform mass spectrometry mode (FTMS) in the full scan ion mode, applying a resolution of 30,000, while acquisition of the mass spectra was performed in every case using the centroid mode. The data-dependent acquisition capability has also been used for MS/MS fragmentation of the three most intense ions of every peak. Data acquisition and analysis have been completed employing Xcalibur 2.1 and MZmine 2.53 [27], respectively.

### 4.11. Statistical Analysis

Correlation of the extract TPC and TFC content with DPPH scavenging capacity was performed in GraphPad Prism 8 (GraphPad Software, San Diego, CA, USA). The correlation coefficient (Pearson’s r) was computed for each pair of variables; the confidence interval was 95% for *p* values, and a heatmap of the correlation matrix was generated. Only *p* < 0.0001 correlations were taken into consideration. PCA analysis was conducted using SIMCA 14.1 software (Sartorius, formerly MKS Umetrics, Umeå, Sweden). Data were Pareto scaled, and the models’ quality was checked through R2X and Q2 cumulative values.

## Figures and Tables

**Table 1:** Codes and %yields of the produced extracts of dry and frozen tepals obtained through MAE and UAE.

Extract Code	Extraction Method	Raw Material	Extract Description	%Yield
CSMWΕfrW	MAE	Frozen	Aqueous extract of frozen tepals by MAE	6.3
CSMWΕfrW/Et			Hydroalcoholic extract of frozen tepals by MAE	6.9
CSMWΕfrEt			Ethanolic extract of frozen tepals by MAE	6.8
CSMWΕdrW		Dried	Aqueous extract of dried tepals by MAE	26.5
CSMWΕdrW/Et			Hydroalcoholic extract of dried tepals by MAE	40.0
CSMWΕdrEt			Ethanolic extract of dried tepals by MAE	39.3
CSUSEfrW	UAE	Frozen	Aqueous extract of frozen tepals by UAE	6.9
CSUSEfrW/Et			Hydroalcoholic extract of frozen tepals by UAE	7.3
CSUSEfrEt			Ethanolic extract of frozen tepals by UAE	8.1
CSUSEdrW		Dried	Aqueous extract of dried tepals by UAE	42.6
CSUSEdrW/Et			Hydroalcoholic extract of dried tepals by UAE	35.0
CSUSEdrEt			Ethanolic extract of dried tepals by UAE	47.8

**Table 2:** Coding and %recovery results of the organic (Org) and aqueous (Aq) fractions obtained using ART.

Code of ART Extract	Origin of Extract	Type of Raw Material	Extract Description	%Yield
CSMWEdrWxadAq	MAE	Dried	Aqueous fraction after ART treatment of dried tepals extract by MAE (CSMWEdrW)	54.4
CSMWΕdrWxadOrg			Organic fraction after ART treatment of dried tepals extract by MAE (CSMWEdrW)	45.6
CSMWEfrWxadAq		Frozen	Aqueous fraction after ART treatment of frozen tepals extract by MAE (CSMWEdrW)	62.7
CSMWEfrWxadOrg			Organic fraction after ART treatment of frozen tepals extract by MAE (CSMWEdrW)	37.3
CSUSEdrWxadAq	UAE	Dried	Aqueous fraction after ART treatment of dried tepals extract by UAE (CSUSEdrW)	60.1
CSUSΕdrWxadOrg			Organic fraction after ART treatment of dried tepals extract by UAE (CSUSEdrW)	39.9
CSUSEfrWxadAq		Frozen	Aqueous fraction after ART treatment of frozen tepals extract by UAE (CSUSEdrW)	60.4
CSUSEfrWxadOrg			Organic fraction after ART treatment of frozen tepals extract by UAE (CSUSEdrW)	39.6

**Table 3:** Extract coding of frozen and dried crocus tepals obtained with MAE, UAE, and ART.

HPΤLC Code	Extract Code	Extraction Method	Raw Material
CSFr1	CSMWΕfrW	MAE	Frozen
CSFr2	CSMWΕfrWxadAq		
CSFr3	CSMWΕfrWxadOrg		
CSFr4	CSMWΕfrW/Et		
CSFr5	CSMWΕfrΕt		
CSFr6	CSUSEfrW	UAE	
CSFr7	CSUSEfrWxadAq		
CSFr8	CSUSEfrWxadOrg		
CSFr9	CSUSEfrW/Et		
CSFr10	CSUSEfrEt		
CSDr1	CSMWΕdrW	MAE	Dried
CSDr2	CSMWΕdrWxadAq		
CSDr3	CSMWΕdrWxadOrg		
CSDr4	CSMWΕdrW/Et		
CSDr5	CSMWΕdrΕt		
CSDr6	CSUSEdrW	UAE	
CSDr7	CSUSEdrWxadAq		
CSDr8	CSUSEdrWxadOrg		
CSDr9	CSUSEdrW/Et		
CSDr10	CSUSEdrEt		

**Table 4:** TPC and TFC of aqueous (including fractions obtained through ART), hydroalcoholic and ethanolic extracts of dried and frozen tepals.

Extract Code	Extraction Method	Raw Material	Total Phenolic Content (TPC)		Total Flavonoid Content (TFC)	
			mg GAE/g ext.	STDEV	mg QUE/g ext.	STDEV
CSMWΕfrW	MAE	Frozen	53.0	1.0	27.7	1.6
CSMWΕfrWxadAq			14.0	0.1	0.0	0.1
CSMWΕfrWxadOrg			119.9	1.4	100.9	1.9
CSMWΕfrW/Et			67.9	0.8	30.4	2.1
CSMWΕfrΕt			64.4	0.3	42.4	1.0
CSMWΕdrW		Dried	58.0	0.8	36.9	3.5
CSMWΕdrWxadAq			12.2	0.4	0.0	0.1
CSMWΕdrWxadOrg			120.3	1.4	112.3	4.3
CSMWΕdrW/Et			65.9	0.8	36.8	0.4
CSMWΕdrΕt			60.6	1.9	36.6	0.3
CSUSEfrW	UAE	Frozen	61.7	4.6	22.7	4.9
CSUSEfrWxadAq			18.6	1.1	0.0	0.2
CSUSEfrWxadOrg			83.0	1.4	69.0	2.3
CSUSEfrW/Et			72.3	1.7	36.2	0.7
CSUSEfrEt			62.5	1.9	42.8	0.6
CSUSEdrW		Dried	61.2	3.5	25.4	0.7
CSUSEdrWxadAq			6.9	0.6	0.0	0.1
CSUSEdrWxadOrg			75.1	0.2	49.8	3.2
CSUSEdrW/Et			64.8	1.0	38.0	1.6
CSUSEdrEt			52.6	0.9	32.6	1.5

**Table 5:** Evaluation results of the antioxidant activity of the extracts of frozen and dried crocus tepals obtained from the treatment with different techniques.

Extract Code	Extraction Method	Raw Material	%DPPH Inhibition			
			200 μg/mL		100 μg/mL	
			Average	STDEV	Average	STDEV
CSMWΕfrW	MAE	Frozen	11.5	0.2	5.2	0.6
CSMWΕfrWxadAq			2.9	0.5	0.4	0.8
CSMWΕfrWxadOrg			36.7	0.1	20.0	1.4
CSMWΕfrW/Et			14.2	0.6	4.2	8.3
CSMWΕfrΕt			10.5	0.3	0.6	0.7
CSMWΕdrW		Dried	15.8	0.2	7.8	0.2
CSMWΕdrWxadAq			4.2	0.3	0.6	0.5
CSMWΕdrWxadOrg			35.8	0.4	16.9	0.1
CSMWΕdrW/Et			17.8	0.6	7.4	1.0
CSMWΕdrΕt			11.4	1.2	6.7	3.7
CSUSEfrW	UAE	Frozen	21.2	1.1	7.9	0.1
CSUSEfrWxadAq			5.3	0.5	1.0	0.4
CSUSEfrWxadOrg			37.0	0.5	16.5	0.5
CSUSEfrW/Et			19.3	1.3	7.6	3.0
CSUSEfrEt			21.2	1.5	10.4	0.9
CSUSEdrW		Dried	13.3	0.5	6.1	0.2
CSUSEdrWxadAq			2.9	0.7	2.6	0.9
CSUSEdrWxadOrg			31.6	0.9	14.9	0.8
CSUSEdrW/Et			14.7	0.6	5.2	0.8
CSUSEdrEt			5.6	1.1	0.2	0.5

**Table 6:** Aqueous extracts and the corresponding organic and aqueous fractions obtained after the treatment of frozen crocus tepals with ART at pilot and lab scale.

HPTLC Code	Extract Code	Extraction Method	Scale	Extract Description
CSA	CSMWΕfrWPL	MAE	Pilot scale	Aqueous extract of tepals by MAE in pilot scale
CSΒ	CSMWΕfrWPLAq			Aqueous fraction after ART treatment of tepals extract by MAE (CSMWΕfrWPL) in pilot scale
CSC	CSMWΕfrWPLOrg			Organic fraction after ART treatment of tepals extract by MAE (CSMWΕfrWPL) in pilot scale
CSD	CSMWΕfrW		Lab scale	Aqueous extract of frozen tepals by MAE in lab scale
CSE	CSMWΕfrWxadAq			Aqueous fraction after ART treatment of tepals extract by MAE (CSMWΕfrW) in lab scale
CSF	CSMWΕfrWxadOrg			Organic fraction after ART treatment of tepals extract by MAE (CSMWΕfrW) in lab scale
CSG	CSMcfrWPL	Mc	Pilot scale	Aqueous extract of tepals by Mc in pilot scale
CSH	CSMcfrWPLAq			Aqueous fraction after ART treatment of tepals extract by Mc (CSMcfrWPL) in pilot scale
CSI	CSMcfrWPLOrg			Organic fraction after ART treatment of tepals extract by Mc (CSMcfrWPL) in pilot scale
CSJ	CSUSEfrW	UAE	Lab scale	Aqueous extract of tepals by UAE in lab scale
CSK	CSUSEfrWxadAq			Aqueous fraction after ART treatment of tepals extract by UAE (CSUSEfrW) in lab scale
CSL	CSUSEfrWxadOrg			Organic fraction after ART treatment of tepals extract by UAE (CSUSEfrW) in lab scale

**Table 7:** TPC and TFC of aqueous (including fractions obtained through ART) extracts prepared at pilot scale.

Extraction Code	TPC		TFC	
	mg GAE/g ext.	STDEV	mg QUE/g ext.	STDEV
CSMWΕfrWPL	65.2	2.1	32.6	1.3
CSMWΕfrWPLAq	16.5	0.6	0	-
CSMWΕfrWPLOrg	147.2	2.0	114.8	4.9
CSMcfrWPL	59.9	1.7	21.4	0.9
CSMcfrWPLAq	12.4	0.2	0	-
CSMcfrWPLOrg	152.8	0.3	85.9	2.8

**Table 8:** Evaluation results of the antioxidant activity of the aqueous extracts prepared on a pilot scale, as well as of the preparations obtained by treatment with adsorbent resins.

Extract Code	%DPPH Inhibition			
	200 μg/mL		100 μg/mL	
	Average	STDEV	Average	STDEV
CSMWΕfrWPL	13.3	0.3	6.2	1.2
CSMWΕfrWPLAq	3.2	0.8	0.9	0.8
CSMWΕfrWPLOrg	47.5	1.0	26.7	0.6
CSMcfrWPL	15.9	3.6	6.1	0.8
CSMcfrWPLAq	2.6	0.5	0.7	0.8
CSMcfrWPLOrg	37.3	1.2	18.9	1.0

**Table 9:** Chemical profile characterization of *Crocus sativus* L. plant tepal extracts by LC-MS.

No.	Rt	*m*/*z*	Adduct	Molecular Formula	MS2 Fragment Ions	Annotation (Potential Assignment Based on Literature Comparison)	CSMWEfrWxadOrg	CSMWEfrWPLOrg
1	1.02	179.0563/225.0616	[M − H]^−^/[M + FA − H]^−^	CH_12_O_6_		hexoses	y *	y
2	1.04	387.1141	[M − H]^−^	C_12_H_22_O_11_		disaccharides	y	y
3	1.18	471.1345	[M − H]^−^	C_17_H_28_O_15_		trisaccharides	y	y
4	1.2	309.0831	[M + FA − H]^−^	C_11_H_18_O_10_	179, 225	3-Hydroxy-4-butanolide	y	y
5	1.21	281.0878	[M − H]^−^	C_10_H_18_O_8_		bis-pentoses	y	y
6	1.85	341.1088	[M − H]^−^	C_12_H_22_O_11_		bis-hexoses	y	y
7	1.98	331.0800	[M+Na-2H]^−^	C_15_H_18_O_7_	313, 171	cinnamoyl hexoside	y	y
8	2.41	345.0823	[M − H]^−^	C_14_H_18_O_10_	299	methyl-galloyl-glucoside	tr **	y
9	2.43	643.1502	[M-H20-H]^−^	C_34_H_30_O_14_	461, 435, 393, 579	tetrahydroxyflavone-hydroxycinnamoyl-di-acetyl-hexoside (Kaempferol 3-(2″,3″-diacetyl-4″-p-coumaroylrhamnoside)	tr	y
10	2.48	689.1548	[M-H20-H]^−^	C_35_H_32_O_16_	536	biflavanol hexoside (ent-Epicatechin-(2alpha->7,4alpha->8)-epicatechin 3-arabinoside)	tr	y
11	4.84	491.1398	[M + FA − H]^−^	C_19_H_26_O_12_	445, 355, 422	phenolic dihexoside (monotropeoside)	tr	y
12	5.8	657.1666	[M + FA − H]^−^	C_27_H_32_O_16_	495, 477	tetrahydroxyflavanone diglucoside (5,6,7,4′-Tetrahydroxyflavanone 6,7-diglucoside)	tr	y
13	5.99	371.098	[M + FA − H]^−^	C_15_H_18_O_8_	325, 162	coumaroyl glucoside (coumarinic acid-β-D-glucoside)	tr	y
14	6.25	393.1764	[M + FA − H]^−^	C_16_H_28_O_8_	347, 326	terpene hexoside (foeniculoside)	y	tr
15	6.42	345.1188	[M + FA − H]^−^	C_14_H_20_O_7_	299	hydroxyphenyl hexoside (2-(3-Hydroxyphenyl)ethanol 1′-glucoside or 4-Methoxybenzyl glucoside)	y	y
16	6.51	659.1451	[M − H]^−^	C_32_H_31_O_14_	497, 345, 330	methyl anthocyanidin coumaroyl glucoside	y	tr
17	6.59	833.196	[M + FA − H]^−^	C_33_H_40_O_22_		flavonol trihexoside (Quercetin 3-sophorotrioside)	y	y
18	6.87	771.1757/817.2020	[M − H]^−^/[M + FA − H]^−^	C_33_H_40_O_21_	609	tetrahydroxy flavone trihexoside	y	y
19	7.17	445.1707/491.1761	[M − H]^−^/[M + FA − H]^−^	C_20_H_30_O_11_	445, 299	hydroxyphenyl dihexoside (crosatoside B)	y	y
20	7.42	567.116	[M-H_2_O-H]^−^	C_28_H_26_O_14_	544, 521	trihydroxy flavone gallate	y	tr
21	7.62	859.212	[M + FA − H]^−^	C_35_H_42_O_22_	610	tetrahydroxy flavone acetyl dihexoside (Kaempferol 3-glucosyl-(6″-acetylgalactoside)-7-glucoside))	tr	y
22	7.91	641.1342	[M − H]^−^	C_27_H_30_O_18_	316, 461	hexahydroxyflavone-dihexoside (Myricetin 3,3′-digalactoside)	y	y
23	8.12	695.1447	[M − H]^−^	C_30_H_32_O_19_	651, 489	tetrahydroxy flavone malonyl dihexoside	y	y
24	8.18	449.1081	[M − H]^−^	C_21_H_22_O_11_	287, 269,	tetrahydroxyflavone glucoside	y	y
25	8.39	625.1395	[M − H]^−^	C_27_H_30_O_17_	463, 445, 300	tetrahydroxyflavone diglucoside (Quercetin 3-O-sophoroside)	y	y
26	8.61	639.1550	[M + FA − H]^−^	C_27_H_30_O_15_	315, 477	tetrahyhydroxy-dimethoxy flavone diglucoside (Isorhamnetin 3,4′-diglycoside)	y	y
27	8.75	609.1445	[M − H]^−^	C_27_H_30_O_16_	285, 429	trihydroxy flavone dihexoside	y	y
28	9.24	593.1497	[M − H]^−^	C_27_H_30_O_15_	484, 429, 447	tetrahydroxyflavone dihexoside (kaempferol 7-O-neohesperidoside)	y	tr
29	9.34	623.1602	[M − H]^−^	C_28_H_32_O_16_	459, 314, 477, 299	trihydroxy-methoxy flavone dihexoside	y	tr
30	9.35	463.0877	[M-H20-H]^−^	C_21_H_22_O_13_	301	galloylglucosyl acetophenone	y	tr
31	9.45	579.1346	[M − H]^−^	C_26_H_28_O_15_	285	Tetrahydroxyflavonol dihexoside	y	y
32	9.8	697.1603	[M + FA − H]^−^	C_29_H_32_O_17_	651, 610	tetrahydroxyflavonol acetyl diglycoside	y	y
33	9.9	447.0928	[M-H20-H]^−^	C_21_H_22_O_12_	284, 285, 327	(epi)catechin glucuronide	y	y
34	10.1	533.1872	[M − H]^−^	C_23_H_34_O_14_	489, 284	tetrahydroxy flavonol malonylglucoside (Kaempferol 3-O-(6″-malonyl-glucoside))	y	y
35	10.77	859.3213	[M + FA − H]^−^	C_38_H_54_O_19_		crocin 2	tr	y
36	10.85	489.1030	[M-H20-H]^−^	C_23_H_24_O_13_	258, 445	tetrahydroxy flavonol acetylglucoside (Kaempferol 3-O-acetyl-glucoside)	y	y
37	12.74	697.2692	[M + FA − H]^−^	C_32_H_44_O_14_	651	crocin 3	y	y
38	13.2	285.0402	[M-H]^−^	C_15_H_9_O_6_	285, 229, 151	tetrahydroxyflavanone	y	y
39	13.21	327.2177			229, 211, 291, 309	lineolic acids derivatives	y	y
40	13.95	329.2334	[M − H]^−^	C_18_H_34_O_5_	229, 211, 293, 311	fatty acid	tr	tr

* yes, ** in traces.

## Data Availability

The original contributions presented in the study are included in the article; further inquiries can be directed to the corresponding author.
